# Land Tenure and Green Production Behavior: Empirical Analysis Based on Fertilizer Use by Cotton Farmers in China

**DOI:** 10.3390/ijerph18094677

**Published:** 2021-04-28

**Authors:** Hui Mao, Yujia Chai, Shaojian Chen

**Affiliations:** 1 Northwest Institute of Historical Environment and Socio-Economic Development, Shaanxi Normal University, Xi’an 710119, China; maohui@snnu.edu.cn; 2 School of Statistics and Big Data, Henan University of Economics and Law, Zhengzhou 450000, China; chaiyj1999@126.com

**Keywords:** land tenure stability, risk preferences, agricultural green production

## Abstract

Stable land rights can increase farmers’ expectations regarding the future and encourage their adoption of green production methods, which is an important guarantee for promoting the development of green agriculture development. This paper takes the fertilizer use as an example and systematically investigated the impact of land tenure stability on the green production behavior of heterogeneous farmers based on a field survey data of 349 cotton-planting farmers from Xinjiang, China. Furthermore, this research aims to assess the differential impact of land tenure stability on different risk preferences, organizational forms and ethnic groups. This study is a continuation of previous studies on factors influencing green production behavior. The results show that land transfers have an inhibiting effect on farmers’ green production behavior and this effect is more significant among risk-averse farmers, local farmers and minority nationalities farmers. The land tenure period can promote the green production of farmers and alleviate the restraining effect of land transfers on farmers’ green production behavior. Additionally, farmers of Xinjiang Production and Construction Corps (XPCC) and large-scale households are more inclined to green production. The Chinese Government needs to further promote land transfer to large-scale households, improve the stability of land rights and adopt differentiated policies for heterogeneous farmers to encourage their green production.

## 1. Introduction

Green agricultural production offers a fundamental way to solve the problems of agricultural resource constraints and environment deterioration as well as promote a sustainable development strategy in China [1]. Farmers’ green production can not only improve the rural ecological environment, but can also improve the quality of cultivated land, which has considerable environmental and economic benefits [2,3,4,5]. The green production (Green production means investing more capital and technology to achieve the goal of reducing the input of chemical fertilizers, mitigating soil pollution and fertility decline, protecting the agricultural ecological environment and promoting sustainable agricultural development [6].) in this paper refers to the farmers’ behavior of reducing the amount of chemical fertilizer and adopting the soil testing and formula fertilization technology, which can improve the quality of cultivated land and reduce the application of chemical fertilizer [7,8]. The Chinese Government has established a series of policy documents in order to promote agricultural green production. The Ministry of Agriculture and Rural Affairs of the people’s Republic of China published the “Technical guidelines for agricultural green development (2018–2030)” in 2018 (http://www.gov.cn/gongbao/content/2018/content_5350058.htm, accessed on 2 July 2018), which clearly proposed to establish a technological system for green agricultural production and promoting agricultural supply-side structural reform. In addition, the central government’s No.1 Document proposed accelerating the construction of green ecological agriculture and realizing the sustainable utilization of agricultural resources for six consecutive years starting in 2015. Therefore, it has become urgent to solve the problems of how to form a long-term mechanism of green production and promote agricultural green production.

The available literature has mainly focused on the influence of land tenure stability on farmers’ green production behavior factors, such as land registration [9], property patterns [10], the approach of farmland circulation [11], transfer price [12], transfer objects [13] and contract type [14], on farmers’ green production behavior, etc. However, due to the development of urbanization and the non-agricultural economy, the homogeneous and isomorphic peasant-farmer pattern has been broken, the phenomenon of farmers differentiation is widespread and heterogeneous farmers show obvious differences in green production behaviors [15,16,17]. Based on the above analysis, this paper comprehensively investigates the impact of land ownership stability on farmers’ green production behavior to address the identified gap in the make up for the lack of available literature.

In theory, land tenure stability (Land with long-term usage rights and land tenure confirmed by written contracts can be regarded as land with stable land tenure [18]. Land adjustment is considered to be the main manifestation of the instability of land rights and it has been one of the most important features of China’s current farmland property rights system since the family contract system was implemented.) mainly affects farmers’ green production behavior in the following ways. Firstly, there is the effect of trading gains. Explicit property rights can protect farmers’ land transaction rights, which in turn gives farmers have the ability to maximize the allocation of resources within the scope of property rights constraints and thus obtain the maximum benefits [19]. Such maximization capability gives farmers greater scope to make long-term investments and further prompts them to manufacture in a greenway. The second factor is the direct incentive effect. Stable land tenure can directly increase farmers’ incomes and provide financial support for farmers’ green production [20]. Moreover, stable land tenure can reduce the probability of the loss of agricultural investments. Such security consequently increases farmers’ expected income, prompts farmers to expand their long-term investments and stimulates farmers to adopt green production [21,22]. When making a long-term investment, farmers will consider the availability of future investment income [23]. Green agricultural technology offers intertemporal gains [24] and is irreversible. Thus, when farmland property rights are more stable, farmers face lower risks of recovering their long-term investment costs. Additionally, they will be more willing to make long-term investment decisions [25,26]. The final effect is that of the mortgages. The underestimation of the farmland values caused by the property rights instability farmland mortgage loans unattractive in terms of both the debit and credit. Thus, land rights stability can clarify ownership and make it more likely for farmers to use farmland as a guarantee to obtain agricultural credit [27,28].

Based on the above points and using the survey data of cotton planting farmers based in Changji Hui Autonomous Prefecture and Kashgar Prefecture, Xinjiang, China, collected in October 2019 in Xinjiang, China, this paper systematically investigates the impact of land tenure stability on green production behavior from the perspective of farmer heterogeneity. The fertilizer application behavior of farmers and the adoption behavior of soil testing formula fertilization technology are used as examples of green production behaviors. Compared with previous research, makes the following three main contributions. First, the research perspective is unique. Examining the impact of land tenure stability on farmers’ green production behavior from the perspective of farmer heterogeneity addresses the assumption of farmers’ homogeneity adopted by previous research on the topic. Second, this study expands on the scope of previous studies on farmers’ green production behavior by simultaneously investigating the impact of (1) whether or not farmers receive a transfer of land, (2) tenure period on farmers’ green production behavior. Third, the sample selected has regional characteristics. This paper focuses on the national features and Corps characteristics of Xinjiang and a large number of national minority peasants’ households and Xinjiang Production and Construction Corps (XPCC) farmers are covered in the sample selection. The XPCC is a special large entity transformed from/established by Xinjiang regional forces of the Chinese People’s Liberation Army in 1954 so as to promote local economic development in Xinjiang. Thus, the research sample has more regional characteristics and pertinence than studies conducted in other areas of China.

## 2. Theoretical Analysis and Hypotheses Development

### 2.1. Farmland Circulation and Farmers’ Green Production Behavior

Property right theory states that unstable property rights will dissuade farmers from making long-term investments [29,30]. In contrast, stable farmland property rights can increase farmers’ future expectations and stimulate green production [31], which is the key to realizing the allocation of production factors and promoting sustainable agricultural development [32]. Farmland circulation will affect farmers’ green production behavior in several ways:

First, trading gains will have an effect. Property rights stimulate farmers to adopt the technology by facilitating transactions and reducing uncertainty [33]. Compared with non-circulating land, farmland with formal circulation is more stable and transactional, so that the effect of farmland trading gains can encourage farmers to invest more in the farmland and stimulate their green production [34,35]. In addition, stable land rights are conducive to farmers’ participation in the farmland circulation market, thereby improving the allocation of the agricultural means of production via the market mechanism. This in turn increases the possibility of farmers realizing the value of farmland investment, consequently stimulating them to make long-term investments and adopt green agricultural technologies [36,37].

The second way farmland circulation affects farmers’ green production behavior is through the direct incentive effect. Contract theory holds that stable contracts can stimulate farmers’ long-term investment by increasing their expected income [38]. For example, Besley (1995) [29] pointed out that stable farmland property rights have a significant incentive effect on farmers’ behavior in terms of increasing long-term investment. In contrast, unstable farmland property rights increase the risks of making long-term farmland investments and reduce farmers’ stability. Accordingly, their confidence of making a return from any long-term investments declines, which reduces their incentive to make long-term investments in farmland [32]. Accordingly, farmers will eschew the green production mode despite its potentially higher future returns in order to reduce the loss of farmland investment returns when they anticipate it is difficulties in realizing the value of farmland investment [26,39]). Furthermore, property rights stability can improve the per capita incomes of peasant households [14], which can further induce farmers to adopt green production. Green agricultural technology has a long cycle before its effects are fully realized. It also incurs more costs in the later stages [40], so the increase in income will provide financial support for farmers to continuously adopt green agricultural technology and stimulate their green production.

The third effect is that of mortgages. Unstable property rights easily lead to the underestimates of the farmland value. Such low valuations make farmland mortgage loans unattractive for both debit and credit. Conversely, land rights stability can clarify ownership and make farmers more likely to use farmland as a guarantee to obtain agricultural credit [41]. Stable land rights can also increase farmers’ ability to obtain credit via mortgaging their own farmland management rights, which helps to provide sufficient agricultural credit funds for agricultural production and boost their ability to make long-term investments in their land [35].

The fourth way farmland circulation affects farmers’ green production behavior is through the crowding-out effect of cost. In the current farmland circulation context, land rent accounts for a high proportion of farmers’ total production costs. Thus, increased costs for transferred land compared with self-owned land will squeeze the agricultural investment cost [42]. The high rent of farmland circulation not only increases the production costs, but also increases the farmers’ profit pressure. Green agricultural technology has a long cycle and high upfront investment costs [40]. If farmers are facing a funding squeeze due to farmland circulation costs or pressures, farmers who carry out farmland circulation may no longer have sufficient funds to invest green agricultural technology in the inflow plots. Based on the analyses above, this paper offers the following hypothesis:

**Hypothesis** **1** **(H1).** *Farmland circulation has a restraining effect on farmers’ green production behavior.*

### 2.2. Tenure Period and Farmers’ Green Production Behavior

The right to use the land for long periods of time is the response of land tenure stability in the time dimension. The longer the tenure period is, the more stable farmers’ expectation of long-term investments will be and they are more likely to make long-term investments and adopt green agricultural technology [43]. Long-term and stable land tenure can improve farmers’ behavior expectations and help them to recover investment costs and obtain benefits from long-term agricultural investment, which has an incentive effect on farmers’ long-term investment [44,45]. Studies have shown that short-term contracts will induce farmers to engage more in short-term production behavior, which is not conducive to farmers’ long-term land investment [46]. Therefore, a longer tenure period provides a time guarantee that incentivizes farmers to adopt green agricultural technology and obtain their intertemporal gains.

Secondly, a long-term and stable tenure period reduces the transaction and the opportunity costs of land assets [47]. Shorter or uncertain land contract periods tend to cause farmers to engage in short-term agricultural production behaviors [48]. For example, Williamson (1996) [49] proposed that long-term contracts have a stronger governance effect than short-term contracts. The longer the tenure period, the greater the number of specific assets in which farmers invest. Shorter or uncertain tenure periods increase farmers’ uncertainty regarding future income, which tends to cause predatory operations on the land and inhibit green production [29]. Pender and Kerr (1998) [36] found that a short-term farmland transfer period among farmers in semi-arid areas of India inhibited farmers’ adoption of green agricultural technologies.

Thirdly, for farmland transfers with different contractual arrangements, long-term contractual relationships are better able to restrain the behaviors of the transfer objects better and guarantees the transferee’s rights to use the land and, thus, boost income expectations [50]. Where short-term farmland circulation is widely spread in China, the land tenure of the transferred land is highly uncertain. Therefore, farmers will reduce their long-term investments in the land in order to minimize possible future losses [51]. For example, Markussen and Tarp (2014) [23] found that a short-term farmland transfer period among farmers had an inhibiting effect on farmers’ investment in steps to increase long-term soil fertility. A longer tenure period is more conducive for incentivizing farmers to make long-term investment in the transferred land to improve soil quality and farming conditions, which increases the possibility of long-term investment returns [9]. This leads to the next hypotheses:

**Hypothesis** **2** **(H2).** *A longer tenure period promotes farmers’ green production behavior.*

## 3. Methods

### 3.1. Empirical Model

Based on previous research, we examine the impact of land tenure stability on the farmers’ green production behavior under the premise of distinguishing the heterogeneity of farmers to test the hypothesis proposed above. The regression equation of farmers’ green production is assumed as follows:

*Y*_1_ = *a*_1_ + *b*_1_ · *Tenure* + *d*_1_ · *X* + *µ*_1_ (1)

In Formula (1), *Y*_1_ is the explained variable, which is measured by the fertilizer application behavior of farmers, including the amount of fertilizer applied by farmers and green agricultural technology (soil testing and formula fertilization technology). Tenure is the core explanatory variable, which is represented by whether farmers transfer land or not and the term of land ownership. *X* represents other explanatory variables. According to the existing research and theoretical logic, the characteristics of farmers and management characteristics are selected as the control variables, including age, gender, education, planting experience of the household head, household size, whether minority nationalities, fertility of cotton fields over the years, etc. *µ*_1_ is a random disturbance term.

### 3.2. Study Area

**Figure 1:**
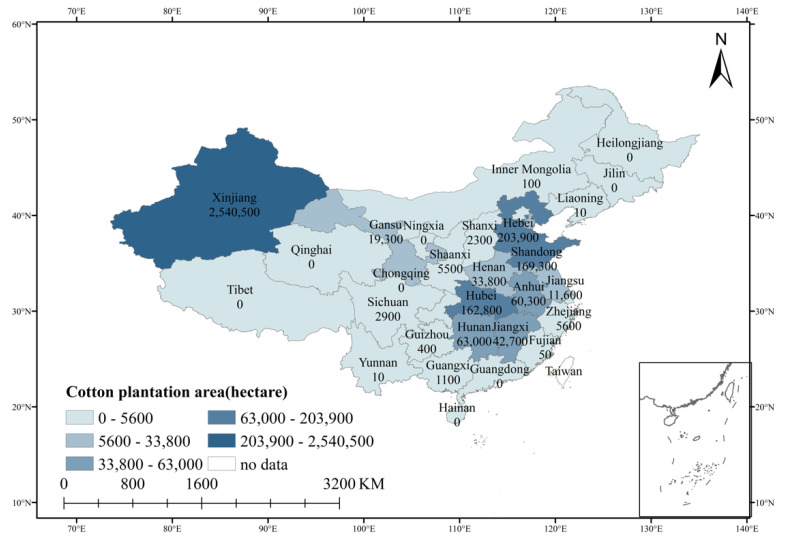
Cotton planting area in each province in China (The source of the data for making the map is “China Rural Statistical Yearbook”).

The main reasons for selecting cotton farmers as our research object are as follows. First, under the background of household contract management in China, agriculture develops as the mode of high input, high yield as well as high resource and environmental costs, which has led to the increasingly serious degradation of cultivated land quality in China. Of the 0.333 billion acres of arable land surveyed and assessed nationwide in 2018, the average quality of arable land was 9.96 and the accumulative area of medium land accounted for 52.72%. The quality of arable land in the western region was lower than the national average. Second, there is a serious overuse of fertilizer in cotton cultivation. The amount of fertilizer input per acre, the amount of fertilizer conversion per acre and the cost of pesticide are all higher than the average amount of the three main grains. Third, in Xinjiang, agricultural moderate scale management level is high. The operating scale of cotton farmers is generally large. The land circulation phenomenon is more common. By the end of September 2020, the total land circulation area in Xinjiang is as high as 1.756 million acres. Fourth, Xinjiang is a high-quality and high-yield cotton region in China. Data from the National Bureau of Statistics shows that Xinjiang’s cotton sown area reached 2,540,500 hectares in 2019 (Figure 1), accounting for 76% of the country’s planted area. Cotton plays an important role in Xinjiang’s agricultural management. Therefore, it is of great research value to take cotton producing areas in Xinjiang as the research object to explore the impact of land tenure stability on fertilizer application and farmers’ green agricultural technology adoption behavior.

**Figure 2:**
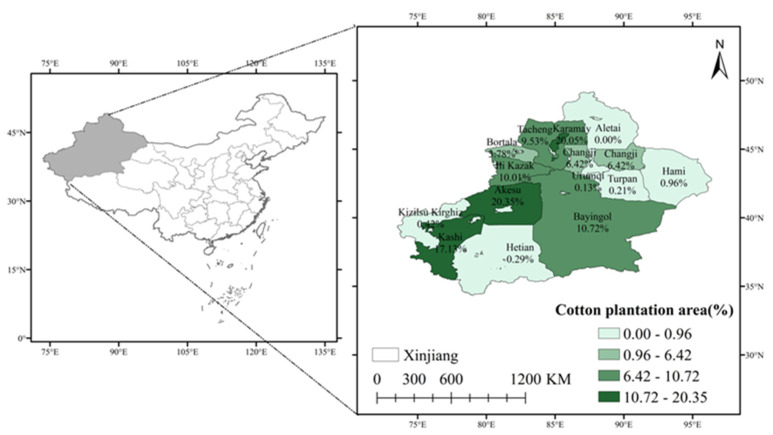
The proportion of cotton area planted in each city to the area of Xinjiang (The source of the data for making the map is “Xinjiang Provincial Statistical Yearbook”).

**Figure 3:**
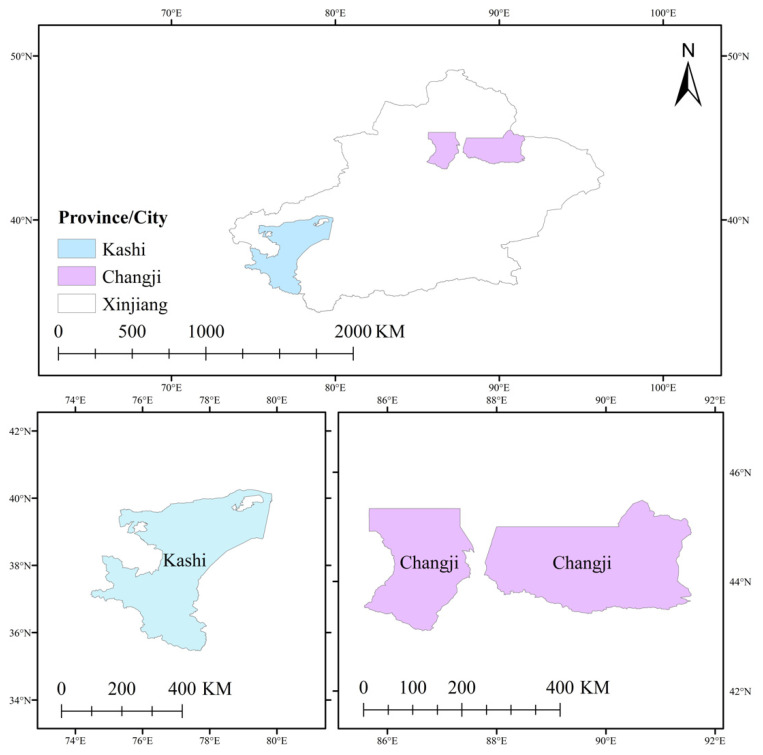
Field experiment sites.

To select samples, we first choose local cotton planting areas. Then, we conducted a survey in Xinjiang Uygur Autonomous Region to carry out systematic sampling in the method of combining stratification and random sampling according to cotton output (Figure 2). First of all, in order to reduce data errors as much as possible and improve the authenticity and effectiveness of the survey data, relevant experts were invited to conduct relevant training for the investigators before the formal investigation. In addition, we recruited bilingual graduate students from Kashgar University to conduct the survey in southern Xinjiang with a large number of minority nationalities samples for the purpose of reducing the surveyed cotton planting farmers’ misunderstanding of the questionnaire (File S1). Secondly, we carried out a preliminary survey both in the northern and southern regions of the Xinjiang Uygur Autonomous Region in August 2019 before the formal investigation. Thirdly, considering the regional spatial differences and the differences between the corps and the local areas, we sufficiently selected the southern and northern Xinjiang. According to the cotton production order, we further choose two regions and the XPCC (Figure 3). Then, we carried out systematic sampling according to cotton production to select two sample counties in each region and choose two townships in each county, totally choosing 12 samples of villages and towns. The sample towns include the areas within and without cultivated land quality protection policy incentives. The proportion of the sample number of various farmers is the same or close to the overall proportion of the administrative village where the farmers are located. We randomly selected thirty farmers from each sample town and 360 farmers were investigated in total. We collected the basic characteristics, household characteristics, land use, fertilizer application behavior, green agricultural technology adoption behavior and other information of farmers and the data year was 2019. We adopted one-on-one interviews to conduct the survey. After eliminating some missing samples of variables, we obtain 339 effective questionnaires. The effective rate of the questionnaire is 94.17%.

### 3.3. Variables

(1) Explained variable: our explained variable is the green production behavior of farmers, including the amount of fertilizer and the adoption behavior of green agricultural technology (soil testing and formula fertilization technology). The amount of fertilizer applied is represented by the amount of fertilizer per acre and the green agricultural technology is represented by the soil testing formula fertilization technology that replaces the traditional fertilizer [7,52].
(2) Explanatory variable: the stability of property in land is expressed by land circulation and tenure. Land is transferred or not means whether farmers transfer into land. If farmers transfer into land, it equals 1. Otherwise, it equals 0. Compared with transferred land, land ownership is more stable and can better stimulate farmers’ long-term investment [53]. The term of land tenure is represented by the years of the land contract signed. A longer-term land tenure means that land tenure is more stable. Compared with farmers with a short term of land tenure, farmers with a longer term of land tenure are more inclined to green production [54]. Operation scale and risk preferences: the operation scale is measured by the actual cotton planting area of farmers. Due to scale economy, farmers with larger operation scale are more inclined to reduce the application amount of fertilizer and adopt green production [55]. According to Tanaka et al. (2010) [56], we use field experiments to measure farmers’ risk preferences. Green agriculture technology is riskier than traditional fertilizer. Compared with risk-averse farmers, risk preference farmers have a higher risk acceptance. They are more willing to try new things and are more inclined to adopt green production [57].
(3) Control variables: the model includes other variables that affect farmers’ green production behavior, such as householder characteristics, family characteristics, management characteristics, organizational form, soil quality characteristics and other variables [58,59]. The definition and analysis of specific variables are in Table 1.

## 4. Results

### 4.1. Farmland Transfers and Farmers’ Green Production Behavior

The OLS estimates, the effect of land transfers on farmers’ green production behavior, are reported in Table 2. Columns (2) and (4) reveals the impact of land transfers on farmers’ consumption of chemical fertilizers and adoption of green agricultural technology. The results show that the coefficient of land transfers is negative and significant at the 1% level. This indicates that after controlling for other influencing factors, land transfers promote farmers’ fertilizer application behavior and inhibit their adoption of green agricultural technology, which supports Hypothesis 1. The reason is that the property rights are different with two farmland types namely those where farmers have received a transfer or land versus those where they have not. Compared with the transferred land, non-transferable land offers a more stable land tenure. Furthermore, farmers have different attitudes towards the two different land types. For transferred land, farmers are more inclined to obtain more short-term benefits through “over-drafting soil fertility”. However, when it comes to their own land, farmers are farsighted. In order to reduce soil pollution and maintain soil fertility, farmers are more inclined to reduce their use of chemical fertilizers and adopt green agricultural technology (the green agricultural technique in this paper refers to soil testing and formula fertilization technology, the specific alternatives to chemical fertilizers that the interviewed farmers are using and are willing to use are soil testing and formula fertilization technology).

Additionally, our results in Table 2 show that farmers willing to take on high risks are more accepting of potential risks and green agricultural technology; in other words, risk preferences have a significant positive impact on farmers’ green production behavior. For the risk-preferences farmers, high risk means high profit and green production may bring higher income, which can increase farmers’ willingness to adopt green production. In contrast, for the risk-averse farmers, green production means the necessity of risk-taking, including risks stemming from improper use of technology and unstable economic returns, which will reduce their incomes. In order to avoid these risks, risk-averse farmers often follow traditional production methods. This result is consistent with the research conclusions of Just et al. (1978) [60], Knight et al. (2003) [61] and Liu (2013) [62].

Furthermore, our result indicates that the scale of land holdings has a significant positive impact on farmers’ green production behavior. This is consistent with the research conclusions of Atanu et al. (1994) [63]. There are two possible reasons for this result. First, green production with the characteristics of scale economy can only take advantage of large-scale land. Second, large-scale farmers have more financial capital and are better able to take risks. In order to achieve scale economy and maximize long-term profits, large-scale farmers pay more attention to the sustainable use of land in production and operation and are more inclined to adopt green production to achieve scale economy effects.

From the result in Table 2, we can also see that membership in the Xinjiang Production and Construction Corps has a significantly positive impact on farmers’ green production behavior. Here are two reasons. First, the XPCC, which has a leading position in cotton production and management level in China, has realized large-scale planting. Compared with local farmers, farmers belonging to the XPCC are more inclined to reduce the amounts of chemical fertilizers and carry out green production. Second, the XPCC benefits from the unification of government and enterprise. From planting and cultivation through to cotton harvesting, the cotton has a higher standard than that of local farmers, which is more conducive to farmers reducing the amount of chemical fertilizers and adopting green agricultural technology.

### 4.2. Land Transfers and Green Production Behavior of Farmers: Endogeneic Treatment

Due to farmers’ participation in agricultural insurance, their behavior has endogeneity problems. First, from the perspective of the theoretical relationship between land transfers and farmers’ green production behavior, land transfers can affect farmers’ green production behavior to some extent. That is to say, farmers’ green production behavior can only exist as the result of a land transfer; in other words, land transfer will affect farmers’ green production behavior whereas farmers’ green production will not affect their land transfer behavior. However, it will result from endogeneity problems will result from omitting important variables, such as household intelligence quotient (IQ) and intergenerational transmission, from the model. Secondly, factor inputs may influence each other and there is a causal relationship between land transfers and farmers’ green production behavior, which will lead to endogeneity problems. Thirdly, farmers’ green production behavior and land transfer behavior have the possibility of “simultaneous decision-making” to some extent and thus pose the problem of “self-selection” based on their own characteristics and comparative advantages.

Therefore, in order to overcome the potential endogeneity problem, this paper firstly uses the situation of rural land transfer as the instrumental variable of rural land transfer behavior. Theoretically, the situation of land transfer in the township where the farmers are located will directly affect the land transfer behavior of farmers, but will not directly affect the fertilizer application behavior and green agricultural technology adoption behavior of farmers, satisfying the correlation and exogeneity hypothesis. The results can be seen in Table 3. Columns (1) to (2) report the estimated results for the instrumental variables, which are consistent with the baseline results above. In addition, the test of weak instrumental variables was conducted in this paper. The Cragg–Donald Wald F statistic was significantly larger than the critical value of Stock–Yogo’s weak instrumental variables, indicating that the model did not have weak instrumental variables. Moreover, the F statistic of the first stage is 208.89, indicating that the instrumental variable has strong validity. The coefficient of the estimated results of instrumental variables changed slightly, but did not change the previous conclusion, indicating that the research results of this paper are robust.

In addition, Lewbel (2012) [64] uses a method to generate a set of valid internal instrumental variables without recourse to external factors. This paper also refers to Lewbel (2012) [64] and adopts the heteroscedasticity identification strategy to try to establish the instrumental variables of land circulation. This method uses the high-order moments of data to generate a set of internal tools to supplement the less effective external tools. The benefit of combining external and internal tools is that it can improve the validity of estimates, especially for instrumental variables where external validity is difficult to guarantee. According to Lewbel (2012) [64], recognition is achieved under two assumptions. First, the error in the first stage is heteroscedasticity, which can be verified by the Breusch–Pagan test in the analysis. Second, there are covariables independent of the conditional covariance between the first and second-order errors. Columns (3) to (4) in Table 3 report the estimated results using external instrumental variables. The first-stage F statistic eliminates the concern of weak tools. The estimated results are similar to those of the instrumental variables, namely farmers are more inclined to reduce fertilizer input on their own land and adopt green agricultural techniques. These findings support the IV estimation in this paper and further prove the effectiveness of using the situation of rural land transfer as the instrumental variable of the model.

### 4.3. Land Transfers and Farmers’ Green Production Behavior: Differences in Risk Preferences

This paper further investigated the impact of land transfers on farmers’ green production behavior under the condition of risk preferences heterogeneity. The regression results are presented in Table 4. Columns (1) and (2) report the impact of land transfer on the risk-loving farmers’ fertilizer application behavior and green agricultural technology adoption behavior. Columns (3) and (4) report the impact on the risk-aversion farmers. The results show that land transfers significantly inhibit the green production behavior of risk-averse farmers more than that of risk-loving farmers. There are two possible reasons for this difference. First, risk-averse farmers are greatly influenced by traditional planting concepts and have a poor willingness to accept green agricultural technologies. Given the presence of uncontrollable agricultural risks, some farmers may make irrational production and management decisions that seem to deviate from the optimal economy. Green agricultural technology is a kind of productive investment that seeks economic benefits and has certain risks. Its long return cycle and high uncertainty increase the instability of its returns. Additionally, land transfer poses risks for farmers of either risk preference type. Increasing the application amount of chemical fertilizer, maximizing land fertility and recovering recouping profits in the short term are effective ways of avoiding risks and thus meets risk-averse farmers’ desire for risk minimization.

### 4.4. Land Transfers and Farmers’ Green Production Behavior: Corps Differences

This section examines the impact of land transfers on farmers’ green production in the case of considering whether or not the farmers are members of the XPCC. The regression results are presented in Table 5. As can be seen, the inhibitory effect of land transfers on local farmers’ green production behavior is significantly greater than that on farmers in the XPCC. The possible reasons for these results are as follows. First, farmers in the XPCC carry out agricultural production under a two-tier management system of regimental farm unification and division. Compared with transfers to local farmers, land transfers to farmers in the XPCC are more secure. Therefore, compared with local farmers, farmers in the XPCC are more likely to participate in formal and normative land transfers and such land transfer s have a greater level of guaranteed stability. Second, farmers in the XPCC are both enterprise employees of enterprises and farmers; they enjoy medical insurance, social insurance and pension guarantees. This gives farmers certain security when deciding how to deal with possible risks in agricultural production by reducing the risk that they will be unable to maintain their basic living standards due to agricultural production losses.

### 4.5. Land Transfers and Green Production Behavior of Farmers: National Differences

Based on previous studies, we found that national minors are mainly concentrated in the border and remote areas in northwestern and southwestern China (Gustafsson and Shi, 2003) [65]. Due to the remoteness of these areas and the mountainous environment, the ethnic minority areas have low levels of transportation infrastructure and industrial development. Their economies mainly depend on farming and stock breeding, which is lower than the national average [66,67]. The Chinese Government has prioritized these ethnic regions to solve the imbalance in regional development [68]. Paying attention to the production of ethnic minority farmers is of great significance to promoting economic development in ethnic regions, reducing economic differences between regions and maintaining ethnic unity [69].

Therefore, this section examines the impact of land transfers on farmers’ green production in the context of considering national differences. The regression results are shown in Table 6. The results indicate that the negative effects of land transfers on green production are significantly greater for minority farmers than that of Han farmers. Several reasons can explain this finding. First, rural economic development in minority-nationality areas is based on a weak foundation, which is greatly restricted by natural conditions and resources. Minority-nationality farmers find it difficult to acquire green agricultural technologies and new knowledge, which limits their adoption of green agricultural technologies. Additionally, language differences place language barriers in the way of minority farmers’ technology learning process. Because adopting green agricultural technology requires a longer adaptation period and is difficult to adopt, these farmers prefer the traditional input method of chemical fertilizer.

### 4.6. Duration of Land Rights and Green Production Behavior of Farmers

Table 7 examines the impact of tenure duration on fertilizer reduction and green agricultural technology adoption. The results show that a longer land tenure term has a significant promoting effect on the reduction of fertilizer application and the adoption of green agricultural technology, which supports Hypothesis 2. The reason for the result is that the soil testing formula fertilization approach to improving soil fertility is a long-term process, which thus has high requirements for the time span of agricultural management. Unstable and short-term farmland property rights and therefore render farmers unable to receive corresponding compensation when investing in formula fertilizer and farmhouse fertilizer, which reduces their incentive for making such a long-term investment in agriculture. In contrast, a longer land rights term provides a time guarantee for making a long-term investment, giving farmers a more considerable expectation recouping any long-term investment. In addition, in order to avoid the risk of income reductions caused by the declining land fertility, farmers will consider the need to the protection of land fertility and reduce the amount of chemical fertilizer and will therefore prefer to adopt green agricultural technology.

## 5. Discussion and Conclusions

Based on fieldwork data of cotton planting farmers in Xinjiang carried out in 2019, this paper has studied the impact of land transfers and tenure duration on farmers’ fertilizer application and green agricultural technology adoption behaviors and further explored and analyzed the impact of land transfers on the differentiation of heterogeneous farmers. The main research conclusions are as follows. First, currently, land transfers in Xinjiang have a significant promoting effect on the amount of chemical fertilizer applied by farmers, but has a significant inhibiting effect on their green agricultural technology adoption behavior. Specifically, farmers with a land transfer have a lower possibility of carrying out green production. Second, compared with the effect on risk-averse farmers, land tenure stability has a stronger effect on the application amounts of chemical fertilizer applied and green agricultural technology tendency of risk-loving farmers. Third, compared with the effect on farmers in the XPCC, the stability of land rights has a stronger effect on the local farmers’ application amounts of chemical fertilizer and their adoption behavior of green agricultural technology. Fourth, compared with the effect on Han farmers, land rights stability has a stronger effect on minority farmers’ application amount of chemical fertilizer and the adoption behavior of green agricultural technology. Fifth, longer tenures can reduce the amount of fertilizer applied by farmers and promote the adoption of green agricultural technologies. Specifically, farmers are more likely to carry out green production when they have a longer tenure.

This study deepens the understanding of the influence of land tenure term on farmers’ green production behavior from transaction cost theory and property rights theory perspective. This research is a pioneering study in the growing body of research on the impact of land tenure on farmers’ green production behavior. By revealing whether land tenure affects farmers’ behavior, we further discussed green production areas that were discussed. Evidence has been provided that land tenure security increases the probability of farmers engaging in green production behavior. This study expands the body of research on the green production behavior of heterogeneous farmers in conditions of uncertain land tenure and contributes to a more established stream of literature on the determinants of farmers’ green production behavior.

## 6. Future Research

The findings presented in this paper have important and relevant practical implications. Firstly, government departments should speed up the construction of marketized allocation of land elements, improve the establishment and development of relevant land transfer trading platforms and guide the signing and effective performance of standardized long-term farmland contracts, giving farmers more stable land management rights. Increasing farmers’ expectation of earning a good income by making a long-term investment in their land can stimulate their use of green production methods. Secondly, based on farmers’ risk-avoidance characteristics, policymakers need to reduce the risk of green agricultural technology through technical training, demonstrations and assistance, so as to promote farmers’ green production. Thirdly, geographical location, cultural differences and ethnic customs mean that minority farmers may face more obstacles in the adoption of green agricultural technologies. Technical service popularization agencies should carry out extensive training on green agricultural technologies in a planned and targeted way with minority households and assign agro-technical popularization personnel to provide personal and in-depth support in order to promote the understanding and adoption of green agricultural technologies by minority peasant households. Fourthly, the local government should learn from the experience of the XPCC in terms of technology promotion and encourage and support the main body of the agricultural technology socialization service supply to provide high quality and efficient technical guidance for farmers. Establishing a platform for information management of agricultural technology socialized services could help farmers keep abreast of the latest technical information and promote the popularization of green agricultural technology.

However, this study also has certain limitations. Firstly, it only includes survey data from 2019. When analyzing the impact of land tenure stability on farmers, continuous multi-period panel data are more effective. Therefore, follow-up surveys of farmers will be conducted to evaluate the dynamic change process of farmers’ green production behavior. Additionally, this study takes cotton farmers as an example and selects two cities in Xinjiang, namely Kashgar and Changji, as the research sites and the scope of the research is relatively limited. In the future, investigations focusing on other major cotton producing areas in China will be conducted and the research conclusions will be extended to the scope of fertilizer application behavior of farmers nationwide.

## Figures and Tables

**Table 1:** Definition and value range of model variables.

Variable	Definition	Mean Value	Standard Deviation	Minimum Value	Maximum Value
Explained Variable					
Consumption of fertilizers	Application amount of fertilizer in cotton field in 2019 (pound/acre (Refraction))	473.223	186.158	120.447	1084.023
Testing soil for formulated fertilization	The farmer weather adopting soil test formula fertilization technology in 2019 (1 = yes, 0 = no)	0.622	0.486	0	1
Key explanatory variable					
Farmland transfers	The farmer has transferred into farmland (1 = yes, 0 = no)	0.688	0.464	0	1
Tenure period	The actual period of farmland farmer transferred(years)	6.407	4.575	0	30
Scale	The actual area of cotton planting (100 acre)	73.750	238.530	0.527	2635.696
Risk preferences	Risk preferences of the household head (σ)	0.774	0.409	0.05	1.45
Control variable					
Age	Age of household head (years)	50.043	9.631	24	90
Gender	The gender of the head (1 = male; 0 = female)	0.894	0.308	0	1
Education	Years of formal education of farmer	7.977	2.794	0	16
Experience	Years of planting crop	15.33	9.617	0	50
Household size (Persons)	Number of persons in household	4.527	1.629	1	16
Organization	The household has participated in the XPCC (1 = yes, 0 = no)	0.249	0.433	0	1
Fertility of cotton fields over the years	1 = bad; 2 = common; 3 = good; 4 = excellent	2.309	0.759	1	4
Minority nationalities	Whether Minority nationalities (1 = yes, 0 = no)	0.547	0.498	0	1

**Table 2:** Estimated results of land transfers and green production behavior of farmers.

	(1)	(2)	(3)	(4)
	Consumption of Chemical Fertilizers	Consumption of Chemical Fertilizers	Green Agricultural Technology	Green Agricultural Technology
Whether farmers transfer land or not	6.037 ***	5.367 ***	−0.730 ***	−0.683 ***
	(1.352)	(1.365)	(0.169)	(0.176)
Risk preferences	−11.387 ***	−10.452 ***	1.641 ***	1.614 ***
	(1.669)	(1.680)	(0.198)	(0.203)
Scale	−0.116 ***	−0.088 ***	0.039 **	0.031 *
	(0.028)	(0.028)	(0.016)	(0.016)
Age	——	0.052	——	−0.005
	——	(0.077)	——	(0.009)
Gender	——	−0.537	——	0.047
	——	(2.594)	——	(0.241)
Education	——	−0.171	——	0.030
	——	(0.256)	——	(0.028)
Years of planting crop	——	0.071	——	−0.001
	——	(0.078)	——	(0.008)
Household size	——	0.540	——	−0.030
	——	(0.420)	——	(0.050)
Whether farmers in the XPCC or not	——	−5.873 ***	——	0.617 ***
	——	(1.375)	——	(0.196)
Fertility of cotton fields over the years	——	−1.648 *	——	0.074
	——	(0.936)	——	(0.103)
Constant term	40.540 ***	41.114 ***	−0.738 ***	−0.893
	(1.604)	(6.261)	(0.184)	(0.722)
Observation	349	349	349	349
R^2^	0.164	0.232	0.255	0.221

Note: ***, ** and * indicate significant at 1%, 5% and 10% levels, respectively; The parentheses are standard errors. Columns (1) and (2) presents the regression results of the impact of land transfers on farmers’ fertilizer application behavior. Columns (3) and (4) provide the regression results for the impact of land transfers on farmers’ green agricultural technology adoption behavior.

**Table 3:** Endogenous treatment.

	IV Estimation		Lewbel (2012)	
	(1)	(2)	(3)	(4)
	Consumption of Chemical Fertilizers	Green Agricultural Technology	Consumption of Chemical Fertilizers	Green Agricultural Technology
Whether farmers transfer land or not	6.045 **	−0.218 **	8.426 **	−0.376 ***
	(2.466)	(0.089)	(3.297)	(0.122)
Control variable	Yes	Yes	Yes	Yes
Constant term	40.693 ***	0.252	39.217 ***	0.350
	(6.292)	(0.227)	(6.692)	(0.245)
First-stage F-statistic	208.89	208.89	47.483	47.483
Observation	349	349	349	349
R^2^	0.232	0.300	0.222	0.228

Note: *** and ** indicate significant at 1% and 5% levels, respectively; The parentheses are robust standard errors. Farmer characteristics, family characteristics, planting characteristics and other variables are controlled in the model. Columns (1) and (3) presents the regression results of the impact of land transfers on farmers’ fertilizer application behavior. Columns (2) and (4) provide the regression results for the impact of land transfers on farmers’ green agricultural technology adoption behavior.

**Table 4:** Land transfer and farmers’ green production behavior: difference in risk preferences.

	(1)	(2)	(3)	(4)
	Risk Lover		Risk Averter	
	Consumption of Chemical Fertilizers	Green Agricultural Technology	Consumption of Chemical Fertilizers	Green Agricultural Technology
Whether farmers transfer land or not	3.708 *	−0.376	6.956 ***	−0.911 ***
	(1.925)	(0.250)	(2.182)	(0.237)
Scale	−0.071 **	0.031	−0.143 **	0.023 *
	(0.032)	(0.024)	(0.064)	(0.012)
Control variable	Yes	Yes	Yes	Yes
Constant term	32.937 ***	−0.237	26.912 ***	1.622
	(8.426)	(0.940)	(9.967)	(1.032)
Observation	349	349	349	349
R^2^	0.143	0.145	0.142	0.122

Note: ***, ** and * indicate significant at 1%, 5% and 10% levels respectively; The parentheses are standard errors. Columns (1) and (2) present the regression results of the impact of land transfers on the fertilizer application behavior and green agricultural technology adoption behaviors of risk-prone farmers. Columns (3) and (4) provide the regression results of the impact of land transfers on the fertilizer application behavior and green agricultural technology adoption behaviors of risk-averse farmers.

**Table 5:** Land transfer and household green production behavior: corps difference.

	(1)	(2)	(3)	(4)
	Farmers in the XPCC		Local Farmers	
	Consumption of Chemical Fertilizers	Green Agricultural Technology	Consumption of Chemical Fertilizers	Green Agricultural Technology
Whether farmers transfer land or not	−0.634	0.723	7.729 ***	−0.933 ***
	(2.047)	(0.454)	(1.764)	(0.198)
Risk peferences	−10.329 ***	2.799 ***	−10.128 ***	1.347 ***
	(2.345)	(0.514)	(2.229)	(0.227)
Scale	−0.001	0.010	−0.109 ***	0.036 *
	(0.035)	(0.010)	(0.035)	(0.020)
Control variable	Yes	Yes	Yes	Yes
Constant term	29.648 ***	0.321	42.105 ***	−0.701
	(10.001)	(2.328)	(7.149)	(0.761)
Observation	87	87	262	262
R^2^	0.320	——	0.181	——
Wald value	——	46.36	——	56.78

Note: *** and * indicate significant at 1% and 10% levels respectively; The parentheses are standard errors. Columns (1) and (2) present the regression results of the impact of land transfers on the fertilizer application behavior and green agricultural technology adoption behaviors of Farmers in the XPCC. Columns (3) and (4) provide the regression results of the impact of land transfers on the fertilizer application behavior and green agricultural technology adoption behaviors of Local farmers.

**Table 6:** Land transfer and farmers’ green production behavior: national differences.

	(1)	(2)	(3)	(4)
	Minority Nationalities		Han Chinese	
	Consumption of Chemical Fertilizers	Green Agricultural Technology	Consumption of Chemical Fertilizers	Green Agricultural Technology
Whether farmers transfer land or not	9.410 ***	−1.144 ***	2.713	−0.000
	(1.897)	(0.226)	(1.875)	(0.269)
Risk preferences	−9.299 ***	1.193 ***	−11.516 ***	2.565 ***
	(2.410)	(0.256)	(2.291)	(0.383)
Scale	−0.478 ***	0.062 **	−0.016	0.011
	(0.151)	(0.030)	(0.024)	(0.012)
Control variable	Yes	Yes	Yes	Yes
Constant term	39.234 ***	−0.472	45.763 ***	−2.739 *
	(7.667)	(0.861)	(10.498)	(1.424)
Observation	191	191	158	158
R^2^	0.237	0.207	0.222	0.420

Note: **, * and * indicate significant at 1%, 5% and 10% levels respectively; The parentheses are standard errors. Columns (1) and (2) provide the regression results of the impact of land trans-fers on the fertilizer application behavior and green agricultural technology adoption behaviors of minority farmers. Columns (3) and (4) present the regression results of the effects of land transfers on fertilizer application and green agricultural technology adoption of Han farmers.

**Table 7:** Estimated results of land tenure duration and green production behavior of farmers.

	(1)	(2)	(3)	(4)
	Consumption of Chemical Fertilizers	Consumption of Chemical Fertilizers	Green Agricultural Technology	Green Agricultural Technology
Tenure period	−0.861 ***	−0.727 ***	0.113 ***	0.108 ***
	(0.191)	(0.183)	(0.026)	(0.025)
Risk preferences	−8.798 ***	−8.313 ***	1.393 ***	1.398 ***
	(1.714)	(1.732)	(0.206)	(0.210)
Scale	−0.051 **	−0.042	0.011	0.008
	(0.024)	(0.029)	(0.008)	(0.007)
Control variable	No	Yes	No	Yes
Constant term	47.916 ***	46.159 ***	−1.682 ***	−1.474 **
	(1.591)	(6.055)	(0.219)	(0.724)
Observation	349	349	349	349
R^2^	0.198	0.251	0.268	0.297

Note: *** and ** indicate significant at 1% and 5% levels respectively; The parentheses are standard errors. Columns (1) and (2) presents the regression results of the impact of tenure duration on farmers’ fertilizer application behavior. Columns (3) and (4) provide the regression results for the impact of tenure duration on farmers’ green agricultural technology adoption behavior.

## Data Availability

Data are not publicly available.

## Supplementary Materials

The following are available online at https://www.mdpi.com/article/10.3390/ijerph18094677/s1, File S1: Survey Questionnaire for Cotton Planting Farmers (2019).
