# Supplementary material for: Land Tenure and Green Production Behavior: Empirical Analysis Based on Fertilizer Use by Cotton Farmers in China
Source: Int J Environ Res Public Health. 2021 Apr 28;18(9):4677. doi: 10.3390/ijerph18094677 (PMC8124971; doi:10.3390/ijerph18094677)
Supplement: Supplementary file 1 [file ijerph-18-04677-s001.zip › ijerph-1165753-supplementary.pdf]

Survey Date: ________ Serial number: ______________City: __________________
County:_________________ Town: _____________________________
Village: _________________ Name of interviewee: ________________
Mobile phone number:______________ Investigator: ________________________
Latitude of survey location: ______________Longitude: _________________________

Survey Questionnaire for Cotton Planting Farmers (2019)

A. Basic situation of farmers

	Gender	Age	Education level	Members of the Communist Party of China	Village Leaders	Years of Planting Cotton
A01.Head of household						
A02. Respondent						

Gender: 1=male, 0=female 1=yes; 0=no

A03. Whether it is a corps household:_______ （a） Whether it is the poor _______
A04. Whether to join a rural cooperative: _______
（a） Whether to sign a purchase and sale contract with a leading enterprise: _______
A05. The area of farmland that your family operates is________mu;
Among them, （a） self-owned arable land________mu;
（b） Degree of land fragmentation: _________(Very neat=1, neat=2, general=3, finely broken=4, very finely broken=5);
（c）Cotton acreage : ________mu; （d）Cotton farmland mainly belongs to: ________（Corps = 1; Place = 2）；
（e） Cotton rented land area: ________ (mu); （f） Rent: ________Yuan/mu;
（g） Type of lease: __________ （1=oral; 2=written, but not completed by the local government; 3=written, and completed by the local government）；
（h） .Starting time of signing the lease（year）: __________;
（i） The type of contracted land: __________ （1=Government direct contract; 2=Farmer households with self-cultivation land; 3=Land owners）.
A06. The cotton planting decision is decided by: ________ （1=The head of the household ; 2=The head of the household is agreed upon by the couple; 3=The head of the household calls the family members to negotiate）.
A07. How many people in your family: ________; Among them,
（a） the number of old people over 65 is: ________;
（b） the number of children under 18 is_____ （c） the number of annual farmers is: ________;
（b） the number of Non-farm labor is: ________.
A08. Whether there are ethnic minorities in the family? : ________ （a） If yes, which nation （0=Han nationality; 1=Uighur nationality; 2=Kazakh nationality; 3=Mongolian nationality; 4=Hui nationality; 5=Kirgiz nationality; 6=others）.
A09. The type of registered residence of the decision maker is: ________ （1=Local agricultural household

registration; 2=Local corps agricultural household registration; 3=Foreign agricultural household registration; 4=Non-agricultural household registration）.

A10. Total household income: ________ ten thousand yuan; non-agricultural income: ________ ten thousand yuan; land transfer income: ________ten thousand yuan.

A11. Agricultural subsidy income: ________ ten thousand yuan; Among them, cotton target price subsidy income: ________ ten thousand yuan.

Other subsidies including: fallow crop rotation subsidies: ________; soil testing formula fertilization subsidy: ________; organic fertilizer subsidy: ________; straw return subsidy: ________; plastic film recycling subsidy: ________; pater-saving irrigation subsidies: ________; straw return subsidy: ________; green pesticide subsidy: ________; subsidy for deep plowing and loosening: ________(Unit: Yuan/mu).

B. Land use and income structure (All crops in 2019)

Crop	a. Planting area（mu）	b. Net income (yuan/mu)
Crop	——	——
B01. Cotton		
B02. Wheat		
B03. Corn		
B04. Soybeans		
B05. Tubers		
B06. Peanut		
B07. Rice		
B08. Rape		
B09. Red dates		
B10. Walnut		
B11. Vegetables		
B12. Fruit		
B13. Other Crops		

Note: If it is not sold out, fill in according to the expected income; if it is not sold but reserved for other use, fill in the area only, and the income is 0

C. Cotton production and sales

Cotton production and sales in 2019

C01. Irrigation water source (1=Surface water, 2=Groundwater, 3=Melt water from ice and snow, 4=Other (Please specify))	
C02. Irrigation method (1=Surface irrigation (furrow irrigation); 2=Self-pressure hose irrigation under the film; 3=Drip irrigation under the film; 4=Underground drip irrigation; 5=Micro-sprinkler irrigation)	
C03. Cotton field fertility (4 = Excellent; 3 = Good; 2 = Medium; 1 = Poor)	
C04. Cotton yield per mu (kg/mu)	
C05. Total sales (kg)	

C06. Price (yuan/kg)	
C07. Sales channels (1= Company; 2=Cooperative; 3=Ginning factory; 4=Corps; 5=National reserve; 6=Other (specify))	
C08. Damage degree of cotton field (0=none; 1=light; 2=medium; 3=heavy)	

(1) Direct costs (average per mu)		——
C09. Total seeds costs		yuan
C09a. Number of varieties planted		
C09b. Planted varieties (1=insect-resistant; 2=salt-tolerant; 3=drought-tolerant; 4=other (specify))		
C09c. Have you received training on planting varieties		
C10. Pesticide costs (treatment of diseases and insect pests, chemical control, etc.)		yuan
C11. Number of pesticide spraying (times/year)		
C12. Prevention and control of common cotton diseases and insect pests in 2019		——
C12a. Varieties of medicine used to treat cotton aphid (1=acetamiprid; 2=Imidacloprid; 3=pymetrozine; 4=other (specify))		
C12b. Cost of medicine for treating cotton aphids (yuan/mu)		
C12c. Amount of pesticide used to treat cotton aphids (g/mu)		
C12d. Times of medicine used to treat cotton aphids (times)		
C12e. Damage degree of cotton aphids (0=none; 1=light; 2=medium; 3=heavy)		
C13. Herbicide costs (Yuan/mu)		yuan
C14. Mechanical operation costs (including plowing, planting, spreading fertilizer, spraying chemicals, machine harvesting, etc.)		yuan
C15. Drainage and irrigation costs (electricity fee + water fee + management fee)		yuan
(2) Indirect expenses (average per mu)		——
C16. The cost of employing workers		yuan
C16a. The number of days employed by workers		day
C16b. The number of days employed by family members		day
C17. Total amount of chemical fertilizer applied per mu		catty
C18. Number of fertilizer applications per mu		times
Employment in 2019	Long-term (the entire production period)	Short-term (temporary)
C19. Total number of employees		
C20. Number of employees over 65		
C21. Number of employees under 18		
C22. The number of years of education is higher than that of the head of the household		
C23. Number of people who have been farming for more than the head of the household		

Note: Don’t know=-999

D. Cleaner production of cotton

The adoption of cotton production technology in 2019

	Green pesticide	Water and fertilizer integration	Water Saving Irrigation	Deep plowing and deep loosening	Organic Fertilizer	Soil testing and fertilizer	Return -ing straw to the field	Plastic film recycling
D01. Adopt -ion ratio (%)								
D02. Cumula -tive use years (years)								
D03. Wheth- -er to receive training								
D04. Outsour -cing ratio								

D05. The main promotion agency of cleaner production technology? (1=Government promotion; 2=Enterprise promotion).

D06. Will you listen to employees’ suggestions on adopting new technologies? (1=Yes; 0=No).

The mechanization of cotton production in 2019

	Degree of mechanization (%)	Outsourcing ratio (%)
D07. Cultivated land/loose land		
D08. Sowing/Nurturing		
D09. Topping		
D10. Fertilization (including formula fertilizer and organic fertilizer)		
D11. Spraying pesticides (including chemical control)		
D12. Harvest		

E. Cotton insurance purchase situation (2019)

Planting crop insurance purchases in 2019

	Purchase area （mu）	Insurance amount (yuan/mu)	Premium (yuan/mu)	Proportion of government subsidies (%)	Payment starting point (%)
E01. Cotton					

E02. Does the village implement policy-based crop insurance?__________ （1=Yes; 0=No)

(a) Insured percentage in the village?_______ （%）

E03. Do you purchase price insurance?____________ （1=Yes; 0=No）

(a) What is the purchase price?_______(Yuan/mu)

E03a. Is price insurance implemented in the village?__________ （1=Yes; 0=No）

(A) Insured percentage in the village?_______ （%）

E04. Insurance income_____ten thousand yuan, including cotton production insurance income _____ten thousand yuan, Cotton price insurance income_____ten thousand yuan

E05. Awareness of price insurance？ ____________(1=never heard of it; 2=heard of it, don't understand; 3=understand a little bit; 4=understand well)

E05a. Are you willing to purchase price insurance？ __________ (1=Yes; 0=No）

E05b. When the protection level is RMB 16,800/ton, your highest willingness to pay is: ___________(Yuan / ton).

E05c. If you pay a premium of 66 yuan, you think the minimum level of protection is ___________(Yuan / ton).

E05d. Please choose a satisfactory combination from the following options__________

a. 15 yuan per ton, the target price is 13,000 yuan/ton	b. 30 yuan per ton, the target price is 14,000 yuan/ton	c. 45 yuan per ton, the target price is 15,000 yuan/ton
d. 60 yuan per ton, target price 16,000 yuan/ton	e. 75 yuan per ton, the target price is 17,000 yuan/ton	f. 90 yuan per ton, the target price is 18,000 yuan/ton
g. 100 yuan per ton, the target price is 19,000 yuan/ton	h. 110 yuan per ton, the target price is 20,000 yuan/ton	I. 120 yuan per ton, the target price is 21,000 yuan/ton

Trust	Village cadre	Insurance company
E06. Are you related to them? （1=Yes; 0=No）		
E07. Do you trust him？ 1=very distrust; 2=distrust; 3=normal; 4=trust; 5=very trust		
E08. Have you ever had a conflict with him？ （1=Yes; 0=No）		

E09. Assuming the implementation of differentiated cotton insurance, you need to choose between three options. Each table below contains the relevant attributes of the three options A, B, and C. Please choose the insurance attribute you prefer according to your true wishes.

Please select ABC 8 times. Tab block type __________ （1/2/3/4/5）

E09a. Your choice is？ ____________ E09b. Your choice is？ _________

E09c. Your choice is？ ____________ E09d. Your choice is？ _________

E09e. Your choice is？ ____________ E09f. Your choice is？ _________

E09g. Your choice is？ ____________ E09h. Your choice is？ _________

F. Cultivated land protection and fallow crop rotation

F01. What do you think is the most serious problem with your own farmland（Multiple selection, sort the selected problems in descending order of severity）<br>（1=soil salinization; 2=decrease of organic matter and fertility; 3=plastic film pollution; 4=land desertification; 5=soil pollution by heavy metals; 6=no problem;7=other __________）	
F02. Whether to rotate crops？（1=Yes; 0=No）	
F02a. If yes, what kind of crop rotation system does it belong to?<br>1=cotton + spring corn+winter wheat; 2=cotton+alfalfa; 3=cotton+wheat+green manure (oil sunflower, sweet clover); 4=cotton+rice; 5=cotton+licorice; 6=cotton+tomato; 7= Cotton + peanuts; 8=others, specify _______	
F03. Do you fallow or not? （1=Yes; 0=No）	
F03a. If yes, how many years have you fallowed？	

G. Pesticide use and productive waste

G01. Do you use pesticides according to the instructions in your cotton growing？ (1=small amount 2=normal 3=excessive)				
G02. Does the use of pesticides in neighboring areas affect your production?？(1=negative influence, 2=no influence, 3=positive influence)				
Productive waste disposal				
	a. Expired pesticides	b. Residual pesticide	c. Pesticide or fertilizer waste bottle/bag/box	d. Agricultural film
G03. Processing method（See code)				

G03. 1=Throw away at hand 2=Throw away trash cans intentionally 3=Special recycling station for collection and disposal 4=Leave to recycle 5=Incineration 6=Landfill 7=Others _____(please note)

H. Borrowing and credit in 2019

Savings	Amount (ten thousand yuan)		Savings	Amount (ten thousand yuan)	
H01. Time deposit			H02. Demand deposit		
Borrowing channels	a. Amount (ten thousand yuan)	b. Annual interest rate (%)	c. Term (month)	d. Number of guarantees	e. Form of guarantee
H03. Relative					
H04. Friends or acquaintances					
H05.Private lending					
H06. Bank					

e. 1=Land mortgage, 2=Contract mortgage, 3=Other ___

Use allocation of loans obtained in 2019 (ten thousand yuan)						
	Agricultural Production	Family medical	Household daily consumption	Weddings and funerals	House construction	other _____
H10. 1st						
H11. 2nd						
H12. 3rd						

H13. Has the Rural Credit Cooperative ever rated your family's credit rating? _________1=Yes; 0=No.

H14 Is there anyone in your family who is religious? _______1=Yes; 0=No;

H15. Are there any outstanding loans?_______1=Yes; 0=No

H16. Financial science knowledge channel______1=Deposit and loan standard; 2=Wealth management products; 3=Insurance; 4=Financial instruments such as related stocks and bonds; 5=Online payment or online loan

H17. Access to financial knowledge channels: _______1=never obtained; 2=family and friends 3=radio, television, internet, etc.; 4=government; 5=financial institution staff

H18. Should you consider choosing an Internet financial platform if you need a sum of money urgently?_______1=Consider B. Don't consider C. Don't understand

H19. If you want to start a business in a rural area and there is insufficient capital for the business, what will you do?_______1=Don't do it if you don't have enough funds, and don't want to owe your debts; 2=borrow money from relatives and friends; 3=private interest-bearing loans; 4=get loans from rural commercial banks.

I. Risk preferences experiment

I1. For an investment, which of the following would you choose? ____________

1=I tend to choose the more risky investment, which has high risk, large return, and large loss

2=I tend to choose the investment with medium risk, which has medium risk, medium return, and medium loss

3=I tend to choose the investment with less risk, which has less risk, less return, and less loss

I2. The first draw: ________ P3. Second draw: ________ P4. The third draw: ________

I3. Time preferences measurement: ________

Number of groups	Option A	Option B	Option	Number of groups	Option A	Option B	Option
a	Current 10 yuan	60 yuan after one month		b	Current 20 yuan	60 yuan after one month	
c	Current 30 yuan	60 yuan after one month		d	Current 40 yuan	60 yuan after one month	
e	Current 50 yuan	60 yuan after one month					

O. Family facilities conditions

O01. Housing area (square meters)		
O02. How many bedrooms in your house?		
O03. Main fuel to heat water	1=coal; 2=electricity; 3=natural gas; 4=firewood, firewood; 5=charcoal; 6=others (please specify _______)	
O04. Bathing equipment	0=none; 1=solar water heater; 2=electric water heater; 3=other (please specify ___________)	

O05. The number of your home appliances: Refrigerator ____ ; TV ____; Smartphone ____; Computer ____;<br>Whether can go online: yes☐ no☐	
O06. Type of toilet (see footnote)	

O06. Toilet type: 0=no; 1=indoor flushing; 2=indoor toilet (no flushing); 3=outdoor flushing public toilet; 4=outdoor non-flushing public toilet; 5=open concrete pit; 6=open soil Pit; 7=other (please specify __________)
